# Mental health knowledge and classroom experiences of school teachers in Aragon, Spain

**DOI:** 10.3389/fpubh.2023.1171994

**Published:** 2023-06-27

**Authors:** José Manuel Granada-López, Enrique Ramón-Arbués, Emmanuel Echániz-Serrano, Raúl Juárez-Vela, Ana Cobos-Rincón, Pedro José Satústegui-Dordá, Noelia Navas-Echazarreta, Iván Santolalla-Arnedo, Michael Nash

**Affiliations:** ^1^Department of Physiatry and Nursing, Faculty of Health Sciences, University of Zaragoza, Zaragoza, Spain; ^2^Research Group GIISA021, University of Zaragoza, Zaragoza, Spain; ^3^Research Group SAPIENF (B53_23R), University of Zaragoza, Zaragoza, Spain; ^4^Faculty of Health Sciences, University San Jorge, Zaragoza, Spain; ^5^Department of Nursing, Faculty of Health Sciences, University of La Rioja, Logroño, Spain; ^6^Biomedical Research Center of La Rioja, CIBIR, Logroño, Spain; ^7^School of Nursing and Midwifery, Trinity College, Dublin, Ireland

**Keywords:** mental health, mental health services, adolescent, child mental disorder, students, school teachers (MeSH)

## Abstract

**Background:**

Research shows that many mental disorders begin in childhood but are sometimes not diagnosed until later years. School-age children spend much of their time in schools and have daily interactions with school teachers.

**Aim:**

Examine school teachers’ experiences of mental disorders in school going children and adolescents and their associated mental health training needs.

**Method and sample:**

A descriptive cross-sectional study was carried out with teachers in Infant-Primary and Secondary Education-Baccalaureate schools.

**Results:**

A convenience sample of 685 teachers responded to the online survey. Participants worked in both urban and rural areas and in Infant-Primary and Secondary Education-Baccalaureate schools. Over half of participants reported classroom experiences of learner mental disorders such as ADHD, anxiety, conduct disorders or autism. Most participants acknowledged a training need, both in recognition of symptoms of mental disorders and in care resources and processes. However, 80% of respondents reported having not received any training in this regard. Participant preferences for training included face-to-face or hybrid – combined online learning. Participants also considered the management of their own mental health to be deficient, therefore any training should incorporate personal mental health awareness and self-help strategies.

**Conclusion:**

In Aragón (Spain), teachers of children and adolescents with mental disorders, recognize a need for training in the identification of symptoms and other aspects of mental healthcare, such as availability and access to services. Protocols for early identification and referral would promote mentally healthy school environments and reduce stigma which could be a barrier to timely intervention. In addition, any training should include mental health self-care for teachers.

## Introduction

The prevalence of mental disorders in Spanish children and adolescents is between 10–20% (1). Mental disorders in children and adolescents can have an adverse impact on learning at school, as well as other areas of life and childhood development. While the first manifestations of mental disorders may occur at an early age they can continue and have further impact in adulthood (2).

Research in the European Union suggests that 50% of mental disorders in adults begin before the age of 15 and 75% before the age of 18 (3). Furthermore, 10% of students between the ages of 6 and 11 have mental disorders, which would mean that there are an average of 2–3 children with some degree of mental health pathology in all elementary classes (3). Therefore, the school environment is an ideal space for early identification and intervention when mental disorders may become apparent (4).

With such high prevalence, Sparling et al. (5) point out that low-cost community mental health interventions, which seek to improve the health and well-being of children and adolescents, are a key point in the context of the Sustainable Development Goals. Jorm et al. (6) have already discussed programs to increase knowledge and beliefs about mental disorders and how these can help to recognize, manage or prevent them. However, a systematic review of mental health literacy programs for school teachers found their knowledge and confidence of helping children with mental disorders is usually low (7).

On the other hand, physical health disorders in childhood and adolescence are easily detected within school environments. Awareness-raising activities on the importance of physical health are often carried out in schools and may be part of school curriculums. However, despite the prevalence of mental disorders in school settings, the latter is not usually addressed in the school setting with the same frequency (8).

Irarrázaval et al. (9) state that interventions in settings such as schools require a multi-sectorial approach involving medicine, education, psychology, social work and public health, but go on to suggest that involvement of the local community as well is important. The involvement of charities and mental health peer support groups would provide an opportunity to have an ecological approach to early identification and intervention for the most vulnerable children whose mental health may be impacted by the social determinants of mental health such as exposure to violence, social deprivation and poverty.

For Wallander et al. (10), preventive school interventions can improve mental disorders in childhood and adolescence, although they have a limited impact in reducing mental illness. Therefore, school interventions should be implemented as a complement and not as a substitute for other services or care interventions. In addition, there is not enough evidence on which type of training programs are best suited to facilitate detection or what is the best intervention for learner mental disorders at school, but training in mental health for teachers would be an essential component of any program (11).

School teachers are also very interested in improving their knowledge in the early detection and prevention of mental disorders, as well as carrying out collaborative work with mental health professionals and receiving guidance from health care settings (12). Education and training, coupled with closer working with community mental health services, primary care and the voluntary sector would enable school teachers to recognize and target interventions and supports. For example, targeted interventions or supports could be tailored to key school milestones, e.g., starting school, exam times or year changes (13).

Freţian et al. (14) point out that while educational interventions directed at students improve their knowledge of mental health, they do not have the desired success on the effects of stigma or social isolation on those who experience mental disorders. A randomised control trial by Milin et al. (15) concerning a mental health literacy program for Canadian secondary school students, confirmed its applicability by teachers with positive effects on knowledge and stigma in mental health. Research shows that such literacy programs can encourage young people to self-seek mental health support (16, 17), and also allow teachers to identify potential mental illnesses early in their students (18).

On the other hand, adequate training of teachers in identifying symptoms of mental disorders makes them appear more competent in this area, which results in better interventions for those affected (19).

This study investigates the classroom experience of teachers regarding mental disorders in their students and their training needs for early detection and intervention.

### Study methodology

This is a quantitative descriptive study of primary and secondary school teachers’ knowledge and experiences of mental disorders in child and adolescent students. Training needs in this area were also explored as well as attitudes towards training in mental health.

### Survey development

A survey was created to be completed online, replicating the study by Nash and Granada (20) in which similar items were explored in primary school teachers in the Republic of Ireland. This gave the survey content and face validity for use in the similar context of the Autonomous Community of Aragon (Spain). In the current study, the sample was extended to secondary school teachers, to capture experiences with mental disorders which may appear between the end of childhood and adolescence. The questionnaire inquired about experiences of mental disorders in the classroom, knowledge of symptoms and any perceived training needs in the area of mental disorders in school students.

### Sample and access

School teachers from public or subsidized centres teaching from infant and primary to secondary and baccalaureate (student age between 3 to 18 years old) in Aragon, Spain. The directors of the schools were informed, with the collaboration of the Department of Education, Culture and Sports of the Government of Aragon, and they distributed study information via email to all the members of the faculty of said schools. The email informed potential participants about the study, its objectives and contained a link to the online survey. Participation was voluntary and participant information confidential.

### Data analysis

Descriptive statistics were used to analyse sample characteristics and types of mental disorders experienced in the classroom. Frequency tables were used to analyse the 5-point Likert scale questions.

### Ethical considerations and consent

Institutional ethical approval was granted for this study by the School of Nursing and Midwifery Research Ethics Committee at Trinity College Dublin. Survey results were anonymous and confidential, and all data has been processed in accordance with current legislation. Opening the link to the survey led potential participants to an information leaflet explaining the study’s aim and method and making it clear that they could withdraw from it at any time up until submission of responses. If they decided to participate, the next step was for them to tick a consent box, following which they could start completing the survey.

## Results

Six hundred eighty-five (685) teachers completed the online survey. Table 1 describes the characteristics of participants.

**Table 1:** Demographic and employment characteristics of study participants (*n* = 685).

Variable	*n*	%
**Gender**		
Male	164	23.9
Female	521	76.1
**Age (years)**		
21–30	66	9.6
31–40	223	32.6
41–50	235	34.3
51–60	145	21.2
>60	16	2.3
**Years of professional practice**		
0–4	150	21.9
5–9	98	14.3
10–14	145	21.2
15–19	99	14.5
≥20	193	28.2
**Work location**		
Private/concerted* infant primary school	37	5.4
Public infant-primary school	286	41.8
Public secondary school – baccalaureate	338	49.3
Private/concerted* secondary school – baccalaureate	24	3.5
**Geographical location**		
Rural	371	54.2
Urban	314	45.8
**Previous mental health training**		
No	548	80.0
Yes	137	20.0

*Concerted – in Spain this is an educational facility part funded by government grants and fee-paying parents.

### Sample characteristics

There are 14,170 school teachers in Aragon eligible to participate in this study. The sample of 685 is acceptable for a confidence level of 95% and a margin of error of 5%. Teachers working in rural areas, in public schools responded at higher rates, regardless of the years of experience as teachers. The distribution of the sample by gender is similar to that shown by the National Institute of Statistics in Spain (67.9% of women teachers in primary, secondary and high school and 97.7% in infant education).

### Types of mental disorders encountered by respondents

**Figure 1:**
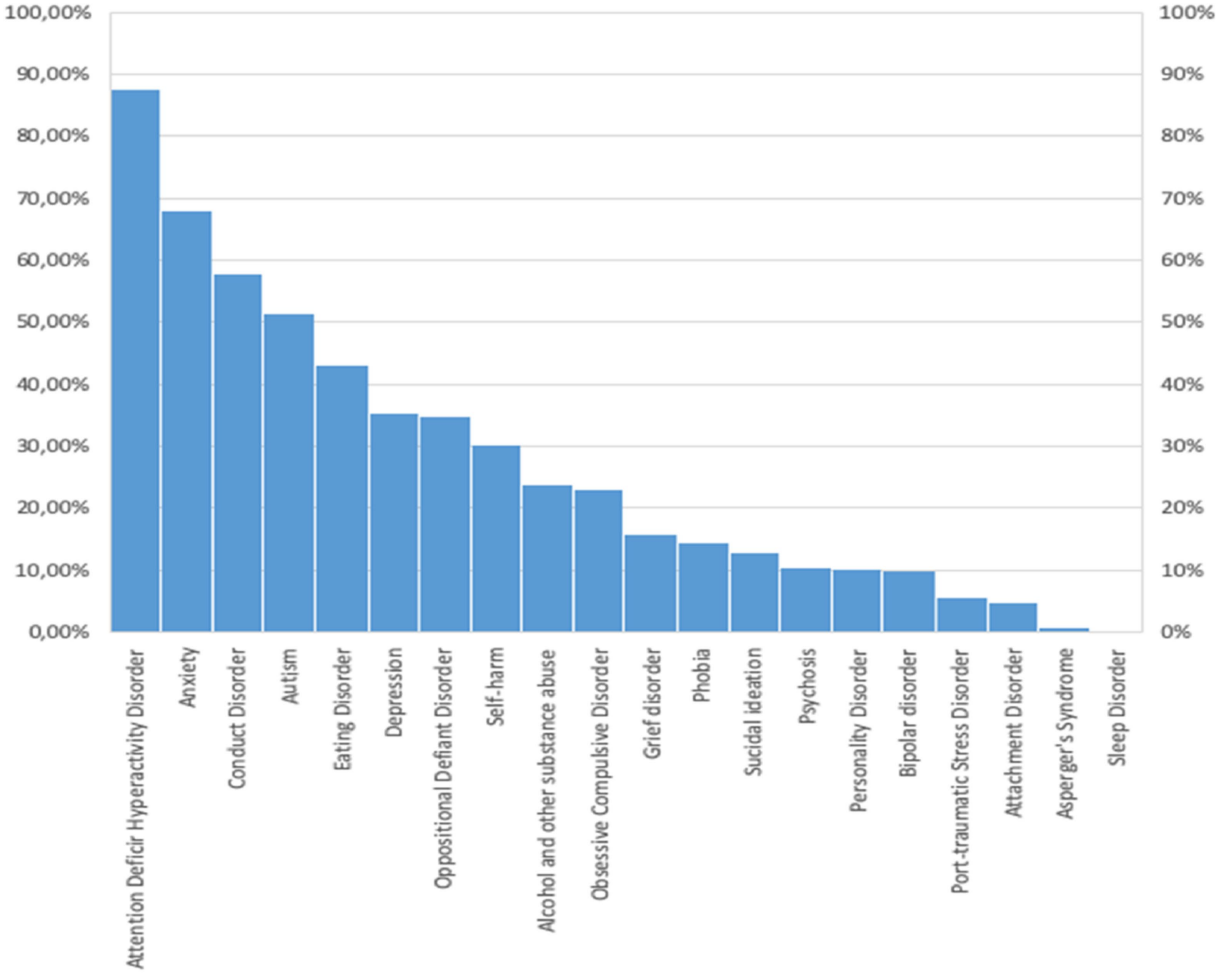
Types of mental disorders reported by teachers in their students.

Mental disorders with high frequencies reported by respondents were Attention Deficit Hyperactivity Disorder (ADHD), Anxiety, conduct disorders and Autism Spectrum Disorders (ASD), which exceeded 50% of the sample. Less frequently reported mental disorders, but not insignificant illnesses, include eating disorders, depression, oppositional defiant disorder, self-harm, substance abuse, and Obsessive–Compulsive Disorder (OCD), all with a frequency greater than 20% (Figure 1).

Serious mental disorders (such as schizophrenia and bipolar disorder) and suicidal ideation appear less frequently, but it should be remembered that this is the teachers’ perception of the disorder and not the actual prevalence of these disorders in schoolchildren.

### Self-assessed knowledge of symptoms of mental disorders and mental health care services

The participants valued their knowledge of symptoms related to mental disorders, as well as about different issues related to mental health care and assistance. To facilitate the assessment of mean and standard deviation, values from 1 to 5 were assigned to the items on the Likert-type scale from “Very bad” to “Very good.” Frequency Tables 2, 3 show the responses.

**Table 2:** Respondents’ self-assessed knowledge of symptoms of mental health issues (*n* = 685).

How would you evaluate your knowledge of…	Very poor *n* (%)	Poor *n* (%)	Neither poor nor good *n* (%)	Good *n* (%)	Very good *n* (%)	Mean ± SD* min.1 – max.5
…symptoms of depression / anxiety	128 (18.7%)	267 (39.0%)	223 (32.6%)	55 (8.0%)	12 (1.8%)	2.35 ± 0.93
…symptoms of ADHD / ASD	69 (10.1%)	189 (27.6%)	300 (43.8%)	108 (15.8%)	19 (2.8%)	2.74 ± 0.94
…symptoms of psychosis / BPD / personality disorder	250 (36.5%)	310 (45.3%)	97 (14.2%)	23 (3.4%)	5 (0.7%)	1.87 ± 0.83
…symptoms of self-harm	246 (35.9%)	276 (40.3%)	124 (18.1%)	34 (5.0%)	5 (0.7%)	1.94 ± 0.90
…symptoms of post-traumatic stress	304 (44.4%)	277 (40.4%)	76 (11.1%)	22 (3.2%)	6 (0.9%)	1.76 ± 0.84
…symptoms of alcohol and other substance abuse	166 (24.2%)	268 (39.1%)	193 (28.2%)	47 (6.9%)	11 (1.6%)	2.22 ± 0.95
…symptoms of suicidal ideation	326 (47.6%)	274 (40.0%)	61 (8.9%)	19 (2.8%)	5 (0.7%)	1.69 ± 0.80
…symptoms of phobia	269 (39.3%)	264 (38.5%)	104 (15.2%)	37 (5.4%)	11 (1.6%)	1.92 ± 0.95
…symptoms of OCD	248 (36.2%)	235 (34.3%)	148 (21.6%)	44 (6.4%)	10 (1.5%)	2.03 ± 0.98
…symptoms of conduct disorder	182 (26.6%)	223 (32.6%)	193 (28.2%)	74 (10.8%)	13 (1.9%)	2.29 ± 1.03
…symptoms of eating disorders	134 (19.6%)	256 (37.4%)	217 (31.7%)	66 (9.6%)	12 (1.8%)	2.37 ± 0.96
…symptoms of attachment disorders	350 (51.1%)	238 (34.7%)	68 (9.9%)	22 (3.2%)	7 (1.0%)	1.68 ± 0.86
…symptoms of grief disorders	279 (40.7%)	250 (36.5%)	116 (16.9%)	30 (4.4%)	10 (1.5%)	1.89 ± 0.93
…symptoms of oppositional defiant disorder	248 (36.2%)	235 (34.3%)	148 (21.6%)	44 (6.4%)	10 (1.5%)	2.09 ± 1.02

*Calculated according to the criteria: very poor = 1, poor = 2, neither poor nor good = 3, good = 4, and very good = 5.

**Table 3:** Respondents’ self-assessed knowledge of mental health services for children and adolescents (*n* = 685).

How would you rate your knowledge of…	Very poor *n* (%)	Poor *n* (%)	Neither poor nor good *n* (%)	Good *n* (%)	Very good *n* (%)	Mean ± SD* min.1 – max.5
…child and adolescent mental health services	282 (41.2%)	269 (39.3%)	96 (14.0%)	30 (4.4%)	8 (1.2%)	1.85 ± 0.90
…medications for use in mental disorders	323 (47.2%)	249 (36.4%)	88 (12.8%)	20 (2.9%)	5 (0.7%)	1.74 ± 0.85
…who to contact if a student has a mental disorder	133 (19.4%)	237 (34.6%)	225 (32.8%)	71 (10.4%)	19 (2.8%)	2.42 ± 1.00
…types of professionals who work in child and adolescent mental health	187 (27.3%)	251 (36.6%)	168 (24.5%)	61 (8.9%)	18 (2.6%)	2.23 ± 1.03
…type of work carried out by child and adolescent mental health professionals	213 (31.1%)	280 (40.9%)	130 (19.0%)	49 (7.2%)	13 (1.9%)	2.08 ± 0.98
…how to write a school protocol/guide on mental health issues in the classroom	354 (51.7%)	243 (35.5%)	60 (8.8%)	22 (3.2%)	6 (0.9%)	1.66 ± 0.84
…how to discuss mental health concerns with parents	247 (36.1%)	285 (41.6%)	113 (16.5%)	35 (5.1%)	5 (0.7%)	1.93 ± 0.89
…how to take care of my own mental health	96 (14.0%)	204 (29.8%)	256 (37.4%)	102 (14.9%)	27 (3.9%)	2.65 ± 1.02

*Calculated according to the criteria: very poor = 1, poor = 2, neither poor nor good = 3, good = 4, and very good = 5.

The knowledge that school teachers self-report as very poor or poor more frequently coincides with those disorders that they identified as less prevalent in their students (attachment disorders, suicidal ideation, severe mental illness and personality disorders, self-harm and post-traumatic stress). Knowledge was reported as better for other disorders such as depression, ADHD, anxiety, conduct disorders, eating disorders, which are more frequently encountered.

Regarding matters related to mental health services and care, participants reported poor, or very poor, knowledge in areas such as medications used in treating mental disorders, writing protocols or guidelines for mental disorders in schools and how to talk to parents or guardians about mental disorders. Participants reported better knowledge of types of professionals who work in child and adolescent mental health and the work that child and adolescent mental health professionals perform.

The previous tables show participants self-reporting high frequencies of low knowledge in many areas, with the exception of participants’ self-reported knowledge about ADHD and ASD. Furthermore, participants’ responses to the category regarding mental health self-care – “how to take care of my own mental health” – indicate a lack knowledge in this regard also. This is something that will need to be borne in mind when developing any mental health care training programs.

### Self-reported training needs on symptoms of mental disorders and mental health care and services

Respondents were asked to select those areas (with the possibility of multiple answers) in which they would like to receive training in relation to the symptoms of mental disorders which were already explored in Tables 2, 3.

Curiously, participants requested more training in areas in which they reported being more knowledgeable and experienced in.

**Figure 2:**
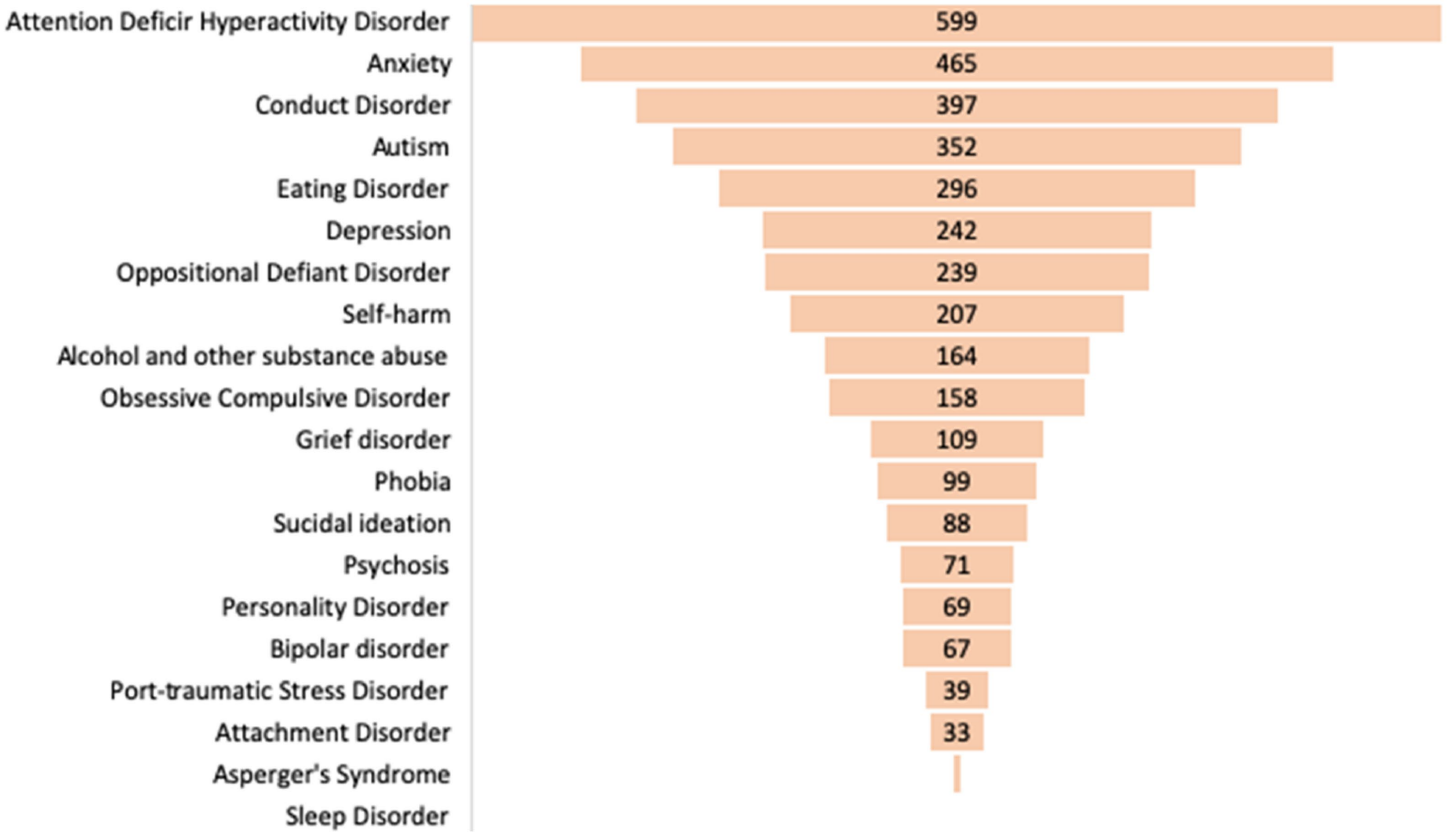
Symptoms of mental disorders that participants reported they would like to receive training.

**Figure 3:**
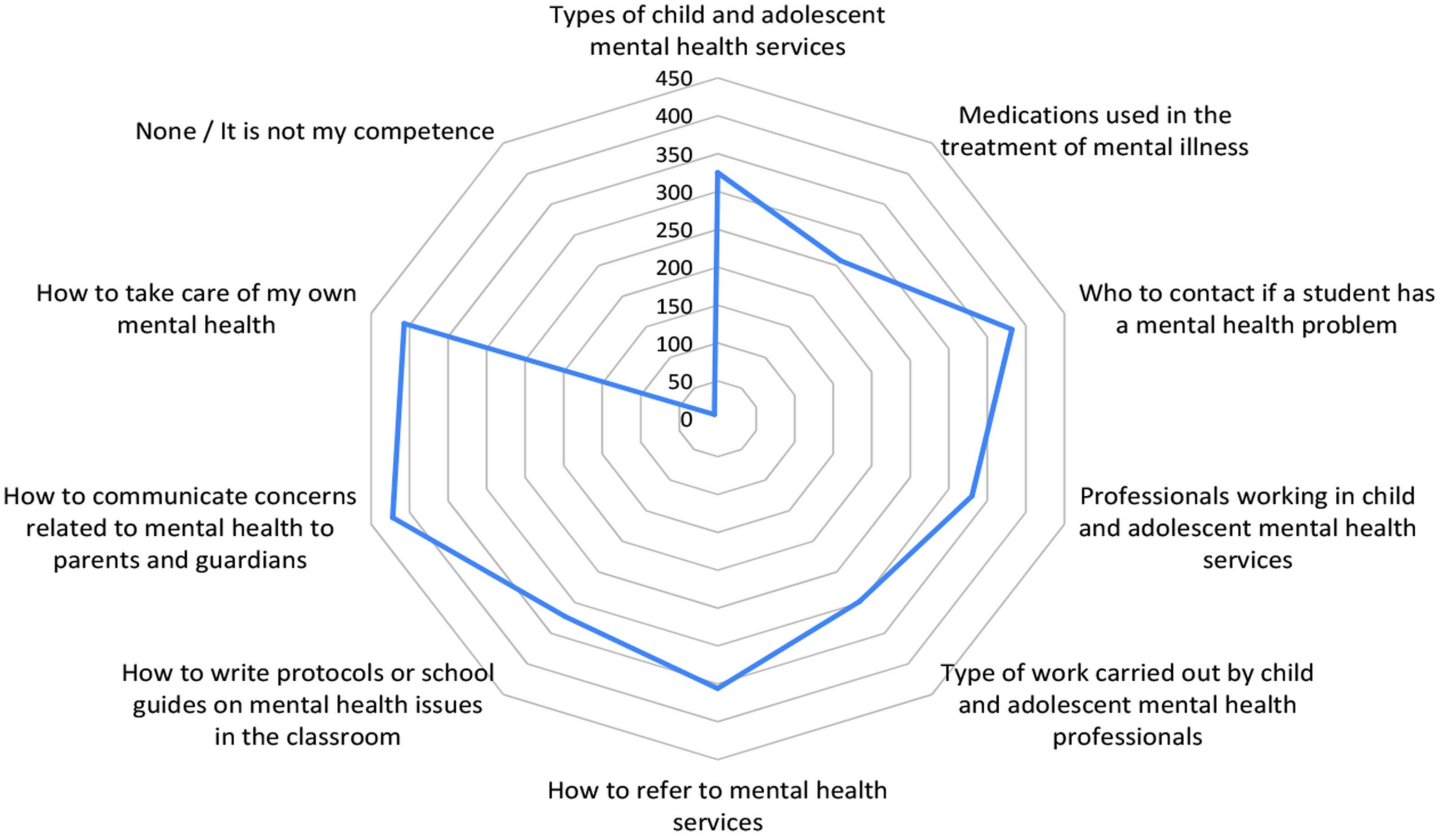
Areas of mental health care and services in which participants reported they would like to receive training.

It is possible that this is the case because participants perceive these disorders (ADHD, anxiety, conduct disorders, eating disorders, and depression), as the most prevalent among their students and so require more knowledge of them. However, participants did not report a need to increase their knowledge in areas such as sleep disorders, Asperger syndrome, attachment disorders, post-traumatic stress, personality disorders, bipolar disorders, schizophrenia and suicidal ideation, items that were indicated by less than 15% of the participants (Figures 2, 3).

### Attitudes towards mental health issues related to their professional role

Teachers were asked about their attitude towards various mental health issues related to their professional role. Using a Likert-type scale participants were asked to record their level of agreement or disagreement (on a five-point graduation from strongly disagree – strongly agree) to predetermined areas. Table 4 summarizes the responses.

**Table 4:** Respondents’ attitudes to mental health awareness training needs in their role (*n* = 685).

Please indicate your level of agreement with the following statements…	Strongly disagree *n* (%)	Disagree *n* (%)	Neither disagree or agree *n* (%)	Agree *n* (%)	Strongly agree *n* (%)	Mean ± SD* min.1 – max.5
I have received adequate training in mental health issues for school-age children and adolescents	228 (33.3%)	324 (47.3%)	50 (7.3%)	69 (10.1%)	14 (2.0%)	2.00 ± 1.00
My teaching center has a standard procedure to know how to handle children and adolescents with mental disorders	141 (20.6%)	199 (29.1%)	190 (27.7%)	140 (20.4%)	15 (2.2%)	2.55 ± 1.10
I have sufficient knowledge to identify a student who may have a possible mental health problem	138 (20.1%)	243 (35.5%)	171 (25.0%)	112 (16.4%)	21 (3.1%)	2.47 ± 1.08
Identifying children and adolescents with mental disorders is not part of my job	306 (44.7%)	220 (32.1%)	94 (13.7%)	50 (7.3%)	15 (2.2%)	1.90 ± 1.03
Most mental disorders ease once students get used to school routines.	331 (48.3%)	226 (33.0%)	104 (15.2%)	20 (2.9%)	4 (0.6%)	1.74 ± 0.86
I need training on mental health knowledge in relation to children and adolescents of school age	24 (3.5%)	31 (4.5%)	79 (11.5%)	266 (38.8%)	285 (41.6%)	4.11 ± 1.01
Helping children and adolescents with mental disorders is not part of my job	369 (53.9%)	198 (28.9%)	74 (10.8%)	35 (5.1%)	9 (1.3%)	1.71 ± 0.94
I would like to receive training on mental health issues in school-age children and adolescents	19 (2.8%)	28 (4.1%)	81 (11.8%)	237 (34.6%)	320 (46.7%)	4.18 ± 0.98

*Calculated according to the criteria: strongly disagree = 1, disagree = 2, neither agree nor disagree = 3, agree = 4, and strongly agree = 5.

**Figure 4:**
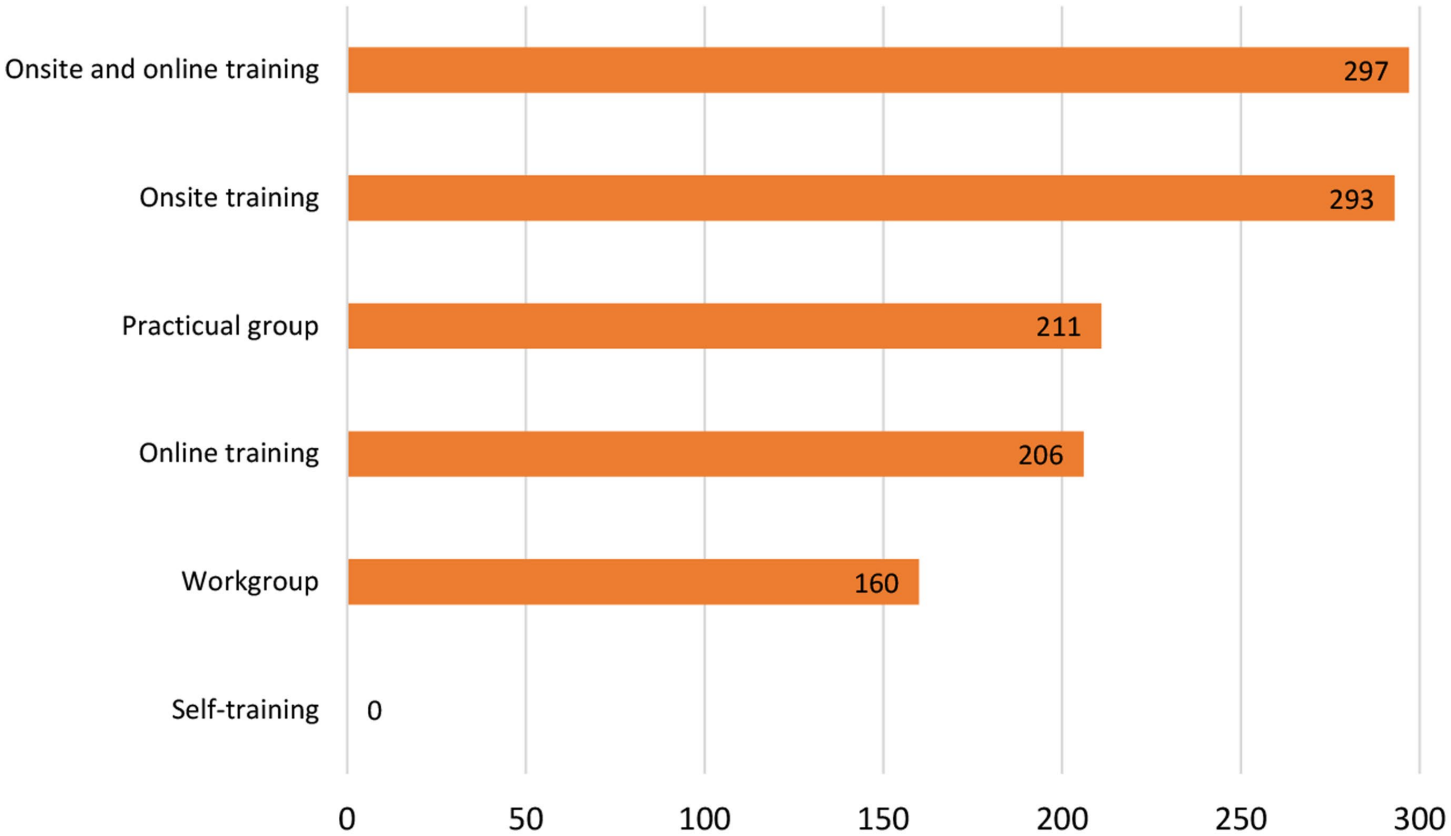
Participants preferred learning methods for mental health training.

**Figure 5:**
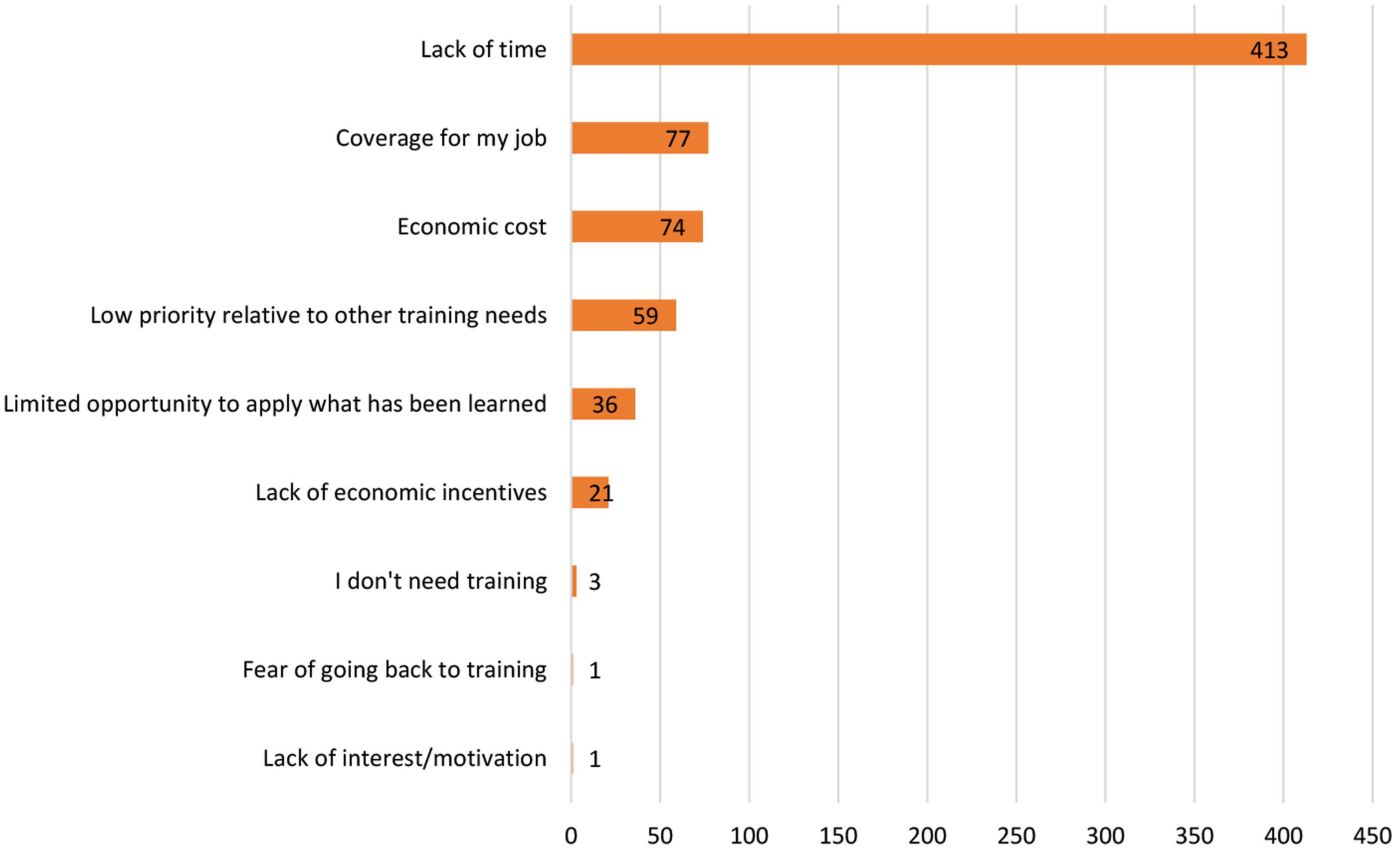
Perceived barriers to training in mental health reported by participants.

Participants display very positive attitudes toward any potential role they may have in relation to helping with mental disorders in school settings. There was a high level of agreement that helping children and adolescents with mental disorders was part of their role and also an acknowledgment of their need for training in relation to mental disorders in children and adolescents of school age. This makes the situation propitious for providing the required training and for the structured implementation of early detection programs and collaboration with mental health care services (Figures 4, 5).

## Discussion

This study explored the mental health care knowledge and training needs of primary and secondary teachers in relation to mental disorders in the classroom including, symptoms and their early detection, knowledge of mental health services and mental health professionals and aspects of mental health care or interventions. The research took place in Aragon, Spain. This will go on to inform the design of training programs and strategies to implement these in school settings. However, a meta-analysis by Yamaguchi et al. (11) suggests inconclusive results on the real impact of such mental health training programs for school teachers on the mental health of students. This included educational programs in the areas of knowledge of mental disorders, attitude towards stigma, confidence to help and possible interventions. Yet, Wang (21) reaffirms the importance of supporting teacher training in areas related to mental disorders due to its impact on the development of children in the school setting.

Participants recognized limitations in their knowledge of symptoms of mental disorders and other aspects related to offering help and support to children and adolescents. However, participants also showed a positive attitude and motivation to attending training, which was also found in research by Nash and Granada (20). Sibanda et al. (22) in their qualitative study point out the difficulties of teachers identifying and managing mental disorders in the classroom, primarily due to lack of training, but also, if school teachers have received training, how to put what was learned into practice. For this reason, joint training with mental health service providers, including the voluntary sector, would increase school teachers’ knowledge and confidence and would allow them to identify first symptoms early and enable them to facilitate the most appropriate onward referral.

Another approach to this issue would be more integration of mental health awareness training into the undergraduate curriculum of school teachers, which has shown good results in terms of increasing their knowledge (16, 17). As teachers, participants in this study already had a university degree, but 80% of them reported not having received specific training in mental health during their university study.

The current study has obtained similar results to a study in the Republic of Ireland by Nash and Granada (20). Although their study had a smaller sample and was located in the area of primary school education, Nash and Granada identified similar school teacher experiences in terms of identifying mental disorders in the classroom, and similar prevalence in areas such as ADHD, anxiety, ASD, conduct disorders and depression. Differences are noted in the area of substance abuse disorders because the Irish sample is from a primary school cohort where, in such a young population, this issue may not yet be manifested. Global epidemiological data shows that up to 20% of children and adolescents suffer from a disabling mental disorder, among which suicide is the third leading cause of death among adolescents and that up to 50% of all adult mental health disorders have their onset in adolescence (23). More specifically, Danielson et al. (24) found childhood mental disorders in one in six children, the most prevalent being anxiety disorders (7.9–11.2%), oppositional defiant disorder (5.7–17.3%) and ADHD (5.1–9.4%).

Participants in this study were clearly in favour of hybrid learning – a mix of in person and online methodologies. However, a study by Hsu et al. (25) which explored the relevance of mental health training for preschool teachers with online methodologies, or in social media networks, concluded that, although it is a methodology appreciated by teachers, it should be developed with caution because multidimensional communicative opportunities could not help school teachers find warm and friendly emotional support to promote their mental well-being. This would suggest training in mental health self-care be facilitated in-person.

A systematic review by Fenwick-Smith et al. (26) provides evidence that mental health promotion programs that focus on resilience and coping skills have positive impacts on the students’ ability to manage daily stressors. These programs can involve weekly class sessions delivered by the teachers themselves and aim to both increase knowledge about mental health and its effects but also increase awareness of mental health resources and supports. This requires teachers involved in school-based programs to have adequate training, especially with programs which prioritise early years, which have been shown to be most effective. In this sense, In this sense, Ford et al. (27) report that school-level variables, variables such as school environment and the quality and character of school life, are factors that promote good mental health. Therefore, there exists the potential to develop school-based indicators of positive mental health which would serve to identify schools that need resources to support the mental health of their students, by improving the school environment.

Of the teachers surveyed, almost 40% state that they have little or very little knowledge of mental health self-care. This is an important finding as the mental health of teachers can influence, and be influenced, by the mental wellbeing of their students. For example, research by Harding et al. (28) on the association of well-being or depression in teachers and their students found better teacher wellbeing was associated with better student wellbeing and lower student distress, whereas higher levels of teacher depression were associated with poorer student wellbeing. Poon et al. (29) suggest that teachers’ stress and sleep problems had negative repercussions on the academic motivation and in-class satisfaction of students. This shows the need to monitor the self-care of teachers’ own mental health when designing training strategies for classroom management of mental disorders. In addition, as Casañas et al. (30) conclude, school based mental health programs must then be aligned with the entire educational community, departments, and teachers, to avoid barriers such as ignorance, fear, or stigma about mental illness.

## Limitations

The use of a convenience sample may represent a sampling bias, in which only those who are most interested in the subject area responded to the survey. The majority of participants were from public schools where there may be a disproportionate level of existing mental disorders. Furthermore, biases typical of anonymous surveys, such as the acquiescence bias (selecting possible options mechanically without much thought) and the social desirability bias (giving socially expected answers and not one’s honest opinion) cannot be ruled out. The survey is not validated, it is an online survey was adapted from the one used by Nash and Granada (20) in the Republic of Ireland. It is a questionnaire designed *ad hoc* to obtain the information of the study.

## Conclusion

This study explored the mental health knowledge and experiences of school teachers in Aragon, Spain. The results of this study confirm that there is a pressing need for training in the area of mental healthcare. Training in areas such as symptom recognition would promote early identification of mental disorders (particularly relating to serious mental illness), which in turn would lead to rapid referral to appropriate mental health services. Participants’ preferred mode of training was hybrid learning – a mix of in person and online classes.

For programs to be sustainable, joint training, between mental health professionals, service providers and teachers, would enable the development of school protocols and referral pathways for mental disorders. Involving parents / guardians and young people in this process would show a commitment to co-production and inclusive learner centred mental healthcare.

Dealing with mental health issues in vulnerable groups, like children and adolescents, can be anxiety provoking, therefore, any training should also include aspects of mental health self-care for teachers, since they consider this an issue that can be improved. The school environment should be a protective place for the mental health of students, but also for that of teachers. The “school years” are an important time for the educational, social and emotional development of children and adolescents. We should endeavour to make them a mentally healthy time as well.

## Data availability statement

The raw data supporting the conclusions of this article will be made available by the authors, without undue reservation.

## Ethics statement

Institutional ethical approval was granted for this study. EAP.2. The patients/participants provided their written informed consent to participate in this study.

## Author contributions

JG-L: conceptualization, software, and writing—original draft preparation. ER-A: methodology. EE-S: validation. RJ-V: formal analysis. AC-R: data curation and resources. PS-D: investigation. NN-E, IS-A, and MN: formal analysis, supervision, and writing—review and editing. All authors contributed to the article and approved the submitted version.

## Conflict of interest

The authors declare that the research was conducted in the absence of any commercial or financial relationships that could be construed as a potential conflict of interest.

## Publisher’s note

All claims expressed in this article are solely those of the authors and do not necessarily represent those of their affiliated organizations, or those of the publisher, the editors and the reviewers. Any product that may be evaluated in this article, or claim that may be made by its manufacturer, is not guaranteed or endorsed by the publisher.
